# CardioRiskNet: A Hybrid AI-Based Model for Explainable Risk Prediction and Prognosis in Cardiovascular Disease

**DOI:** 10.3390/bioengineering11080822

**Published:** 2024-08-12

**Authors:** Fatma M. Talaat, Ahmed R. Elnaggar, Warda M. Shaban, Mohamed Shehata, Mostafa Elhosseini

**Affiliations:** 1 Faculty of Artificial Intelligence, Kafrelsheikh University, Kafrelsheikh 33516, Egypt; fatma.nada@ai.kfs.edu.eg; 2 Faculty of Computer Science & Engineering, New Mansoura University, Gamasa 35712, Egypt; 3 Faculty of Medicine, Mansoura University, Mansoura 35516, Egypt; ahmedrefaatelnaggar@gmail.com; 4 Communications and Electronics Engineering Department, Nile Higher Institute for Engineering and Technology, Mansoura 35511, Egypt; warda_mohammed@nilehi.edu.eg; 5 Department of Bioengineering, Speed School of Engineering, University of Louisville, Louisville, KY 40292, USA; 6 Computers and Control Systems Engineering Department, Faculty of Engineering, Mansoura University, Mansoura 35516, Egypt; melhosseini@mans.edu.eg

**Keywords:** active learning, cardiovascular diseases (CVDs), eXplainable artificial intelligence, risk prediction

## Abstract

The global prevalence of cardiovascular diseases (CVDs) as a leading cause of death highlights the imperative need for refined risk assessment and prognostication methods. The traditional approaches, including the Framingham Risk Score, blood tests, imaging techniques, and clinical assessments, although widely utilized, are hindered by limitations such as a lack of precision, the reliance on static risk variables, and the inability to adapt to new patient data, thereby necessitating the exploration of alternative strategies. In response, this study introduces CardioRiskNet, a hybrid AI-based model designed to transcend these limitations. The proposed CardioRiskNet consists of seven parts: data preprocessing, feature selection and encoding, eXplainable AI (XAI) integration, active learning, attention mechanisms, risk prediction and prognosis, evaluation and validation, and deployment and integration. At first, the patient data are preprocessed by cleaning the data, handling the missing values, applying a normalization process, and extracting the features. Next, the most informative features are selected and the categorical variables are converted into a numerical form. Distinctively, CardioRiskNet employs active learning to iteratively select informative samples, enhancing its learning efficacy, while its attention mechanism dynamically focuses on the relevant features for precise risk prediction. Additionally, the integration of XAI facilitates interpretability and transparency in the decision-making processes. According to the experimental results, CardioRiskNet demonstrates superior performance in terms of accuracy, sensitivity, specificity, and F1-Score, with values of 98.7%, 98.7%, 99%, and 98.7%, respectively. These findings show that CardioRiskNet can accurately assess and prognosticate the CVD risk, demonstrating the power of active learning and AI to surpass the conventional methods. Thus, CardioRiskNet’s novel approach and high performance advance the management of CVDs and provide healthcare professionals a powerful tool for patient care.

## 1. Introduction

Cardiovascular diseases (CVDs) continue to pose a significant global health challenge, accounting for a substantial burden of morbidity and mortality [1]. Timely risk prediction and prognosis play a critical role in identifying individuals at high risk of developing CVDs, enabling early intervention and personalized treatment plans [2].

CVDs are the primary cause of mortality worldwide, resulting in approximately 17.9 million deaths annually. CVDs encompass a range of disorders affecting the heart and blood vessels, such as coronary heart disease, cerebrovascular disease, rheumatic heart disease, and other related conditions. The majority of CVD deaths, specifically over 80%, are caused by heart attacks and strokes. Furthermore, approximately one third of these deaths occur prematurely in individuals who are under the age of 70 [1].

Based on a recent estimate by the World Heart Federation (WHF), the global number of fatalities caused by CVDs has increased from 12.1 million in 1990 to 20.5 million in 2021. In 2021, CVD was the primary cause of death worldwide, responsible for 80% of the fatalities in Low- and Middle-Income Countries (LMICs) [1].

When assessing the CVD risk in clinical practice, the following gold standards are widely used: (i) The Framingham Risk Score (FRS): The FRS is a popular tool for calculating the 10-year CVD risk based on characteristics such as age, gender, blood pressure, cholesterol, and smoking status [3] Although it provides a broad risk assessment, it may not fully capture small changes. (ii) Blood Tests: Measuring lipid profiles such as cholesterol and triglycerides is common practice. Elevated levels of these indicators increase the risk of CVDs. (iii) Imaging Techniques: Various imaging modalities, such as MRIs, CT scans, and echocardiograms, can provide detailed information about the structure and function of the heart [4]. (iv) Clinical Assessment: Risk factors such as diabetes, hypertension, and family history must be evaluated during the cardiovascular risk assessment process. (v) SCORE Models: The European Society of Cardiology (ESC) developed the SCORE (Systematic Coronary Risk Evaluation) models, a set of risk assessment tools that evaluate the 10-year risk of fatal cardiovascular events. These models are intended to help medical professionals to determine a patient’s likelihood of experiencing a cardiovascular event (such as a heart attack or stroke) over the next ten years. In Europe, the SCORE models are widely used, providing separate risk charts for high-risk and low-risk sites [5].

CVDs, including coronary artery disease (CAD), atrial fibrillation (AF), and other cardiac and vascular disorders, remain the leading cause of global mortality. As the living standards improve and the stress levels increase, the incidence of CVDs continues to rise at an alarming rate [6].

Recent estimates [7,8] suggest that, by 2030, CVDs will claim the lives of approximately 23 million people. Myocardial infarction, atrial fibrillation, and heart failure are examples of different types of CVDs [9,10]. The occurrence of CVDs can be influenced by various factors such as race or ethnicity, age, gender, body mass index (BMI), height, and torso length, as well as blood test results evaluating parameters like renal function, liver function, and cholesterol levels [11,12].

Despite their usefulness, the conventional methodologies for assessing cardiovascular risk have significant disadvantages that highlight the need for alternative tactics in artificial intelligence (AI( [13]. (i) Limited Precision: While the traditional methodologies may provide basic risk evaluations, they may be unable to identify individual deviations that are small enough to be precise in detecting complex threats. (ii) Static Risk Variables: The conventional techniques usually consider age, blood pressure, cholesterol levels, and other static risk variables. Regardless of their significance, these variables may not respond swiftly to changing patient needs. (iii) Illusion: While blood tests and imaging technologies provide helpful information, they may not provide a complete and up-to-date picture of the complex interconnections between the variables that influence cardiovascular health [14,15]. (iv) Population-Based Averages: Because the conventional models are based on population averages, they may overlook the changes within specific demographic groups or individual patient characteristics. (v) Complexity of Risk Factor Interaction: The multiple risk variables may interact in various nuanced and nonlinear ways. Traditional models’ anticipated accuracy may be limited because they cannot fully describe the complexity of these relationships. (vi) Inability to Learn and Adapt: The traditional risk assessment approaches are typically incapable of learning and changing over time. As a result, they may fail to update the risk assessments with new or changing patient data [16,17].

Therefore, alternative methods should be sought for the early detection of CVDs, such as AI. AI-based models can potentially improve the precision and personalization of cardiovascular risk assessments due to their capacity to learn dynamically, adapt, and consider a wide range of data sources. AI algorithms have demonstrated significant potential for increasing risk assessment accuracy and prognostic evaluations. These algorithms outperform the traditional statistical models by capturing the complex interconnections and nonlinear interactions between variables and outcomes [18,19].

AI algorithms can examine many different kinds of patient data, like their medical history, imaging results, and genetic information, to find hidden patterns and links that may initiate CVDs and cause them to become worse [20]. By leveraging large-scale datasets and powerful computational techniques, AI models can capture intricate relationships and identify risk factors that traditional statistical approaches may miss.

One promising approach in AI research is the integration of eXplainable AI (XAI) techniques, which aim to provide transparent and interpretable predictions. XAI methods enable healthcare professionals to understand the underlying factors and reasoning behind the risk assessment, instilling confidence in the model’s predictions and facilitating clinical decision-making [21]. This transparency is crucial in healthcare, where interpretability and explainability are paramount for gaining trust and acceptance from medical practitioners [22].

Active learning is another important AI technique that can enhance risk prediction and prognosis models. Active learning algorithms intelligently select informative samples from the dataset for expert annotation, thereby improving the model’s performance with limited labeled data [23]. By actively acquiring new data points that are the most informative for risk assessment, active learning helps to overcome the challenge of data scarcity. It enhances the AI model’s generalization capabilities.

Moreover, attention mechanisms have gained prominence in AI research due to their ability to focus on the salient features and patterns within patient data. By selectively attending to relevant information, attention mechanisms improve the precision and clinical relevance of risk predictions [24]. This can aid in identifying high-risk individuals who may benefit from targeted interventions and personalized treatment plans.

Integrating XAI, active learning, and attention mechanisms in risk prediction and prognosis models holds great promise for improving the accuracy, interpretability, and clinical relevance of CVD assessments. By harnessing the power of AI and leveraging diverse patient data, these hybrid AI models can transform cardiovascular medicine, identifying high-risk individuals and facilitating personalized interventions for improved patient outcomes.

CVD risk assessment remains a crucial yet challenging task in healthcare. The traditional models have played a vital role in this domain, providing valuable insights. However, their limitations in handling complex and diverse patient data, along with the growing need for interpretable AI in clinical settings, necessitate the exploration of more advanced techniques.

This paper addresses this critical need by introducing CardioRiskNet, a novel hybrid AI model for CVD risk assessment and prognosis. CardioRiskNet surpasses the traditional models by harnessing the power of XAI, active learning, and attention mechanisms. This synergistic approach offers several advantages:

- Precision of eXplainable AI (XAI): CardioRiskNet leverages XAI techniques to ensure transparency and interpretability in its decision-making process. These fosters trust and understanding among healthcare professionals who can utilize the model’s insights with greater confidence.
- Adaptability of Active Learning: The model employs active learning to strategically select and learn from informative data samples, enhancing its accuracy and efficiency over time.
- Focus of Attention Mechanisms: By incorporating attention mechanisms, CardioRiskNet prioritizes the relevant features and patterns within patient data, leading to more accurate predictions.

Main Contributions:

This research contributes significantly to the field of cardiovascular risk prediction through the following:

- Introduction of CardioRiskNet: This hybrid AI model establishes a new standard for CVD assessment and prognosis through its comprehensive and innovative approach.
- Attention Mechanism Integration: By focusing on the critical features, attention mechanisms enhance the model’s predictive power.
- XAI Application: XAI techniques ensure transparency in CardioRiskNet’s decision-making, fostering trust among healthcare professionals.
- Superior Performance Metrics: CardioRiskNet outperforms the traditional models by achieving a 98.7% accuracy rate, 99% area under the curve (AUC), and a remarkable F1-Score of 98.7%, along with exceptional sensitivity and specificity.
- Utilization of Real-World Dataset: The model’s effectiveness is demonstrated using a comprehensive Heart Failure Clinical Records Dataset, showcasing its applicability in real-world settings.
- Benchmarking Against the Traditional Methods: A critical evaluation of the existing cardiovascular risk assessment tools highlights the limitations that CardioRiskNet effectively addresses.

The remaining sections of the paper are organized as follows: Section 2 examines the previous algorithms, Section 3 details the methodology, Section 4 presents the results and compares the proposed model with the existing methods, emphasizing the benefits of XAI, and Section 5 concludes the paper with the key findings and future research directions.

## 2. Related Works

Several studies have explored the application of AI in the risk prediction and prognosis for CVDs.

Nirschl [25] focused on developing a Convolutional Neural Network (CNN) classifier to identify clinical heart failure from H&E-stained whole-slide images. The study utilized a dataset of 209 patients, allocating 104 for training and the remaining 105 for independent testing. The CNNs outperformed the conventional feature-engineering approaches on the test set, detecting the patients with heart failure or severe disease with 99% sensitivity and 94% specificity. Importantly, the CNNs outperformed two professional pathologists by approximately 20%. The findings indicate that the deep learning analytics of EMB can be utilized to predict cardiac outcomes.

Moshawrab M et al. [26] systematically reviewed the literature focusing on smart wearable applications for detecting and predicting CVDs. They performed a comprehensive search and analyzed the selected documents to identify the important factors, including the year of publication, recorded vital signs, studied diseases, utilized hardware, employed smart models, datasets utilized, and performance metrics.

Pal M et al. [27] employed two robust machine learning algorithms, specifically Multi-Layer Perceptron (MLP) and K-Nearest Neighbor (K-NN), to identify CVDs using publicly accessible data from the University of California Irvine repository. In order to enhance the efficiency of the models, any data points that deviated significantly from the norm and any attributes that lacked values were eliminated. The empirical findings indicated that the MLP model outperformed the K-NN model, achieving an accuracy rate of 82.47% and an area under the curve value of 86.41%. Hence, the researchers suggested utilizing the MLP model for the purpose of automated detection of CVDs. Additionally, it was proposed that the suggested methodology could be modified to identify other diseases and that the effectiveness of the model could be assessed using various established datasets.

García-Ordás M. T et al. [28] suggested employing deep learning techniques and feature augmentation methods to assess patients’ risk of CVDs. The proposed methods produce better results than other advanced approaches, with a 4.4% improvement and 90% precision. This represents a significant advancement, particularly for a widespread disease affecting a large population.

Chicco et al. [29] investigated the potential of machine learning for predicting heart failure patient survival using a dataset of 299 patients. Their analysis identified serum creatinine and ejection fraction as the two most relevant features for survival prediction. Interestingly, they found that models built using only these two factors achieved higher accuracy compared to models utilizing the entire dataset. This finding highlights the importance of feature selection and exploring the potential for simpler models with high predictive power, which aligns with our focus on interpretability in CardioRiskNet. Table 1 presents the various machine learning and deep learning algorithms for risk prediction and prognosis in CVDs.

From the discussed literature studies and the table above, the research gaps can be summarized in the following points:

- Integration and Holistic Modeling: There is an apparent gap in creating a unified AI framework that cohesively integrates diverse methodologies (CNNs, machine learning, and deep learning) to comprehensively enhance the CVD risk prediction and prognosis.
- Dynamic Learning and Personalization: The existing AI models exhibit limitations in adapting to and learning from real-time individual patient data for personalized risk assessment.
- Model Interpretability: The prevalent black-box nature of many AI approaches, especially in deep learning, poses challenges to clinical adoption due to the necessity for transparent and interpretable decision-making.
- Dataset Dependency: The efficacy of many AI models hinges on the availability of extensive, well-annotated datasets, which may not always be accessible or applicable across all conditions or regions.
- Handling Heterogeneous Data: There is a lack of robustness in AI models towards processing and analyzing data from mixed sources, such as different imaging modalities, wearable devices, and electronic health records, crucial for a comprehensive CVD risk assessment.

This study presents CardioRiskNet, a novel hybrid AI-based model addressing the abovementioned shortcomings. The suggested framework combines eXplainable AI, active learning, and attention mechanism approaches to forecast and prognosticate CVD risk. CardioRiskNet promises to provide reliable, interpretable risk assessments by using cutting-edge AI approaches, ultimately assisting in identifying high-risk people and enabling targeted therapies for better patient outcomes.

## 3. The Proposed Methods

**Figure 1:**
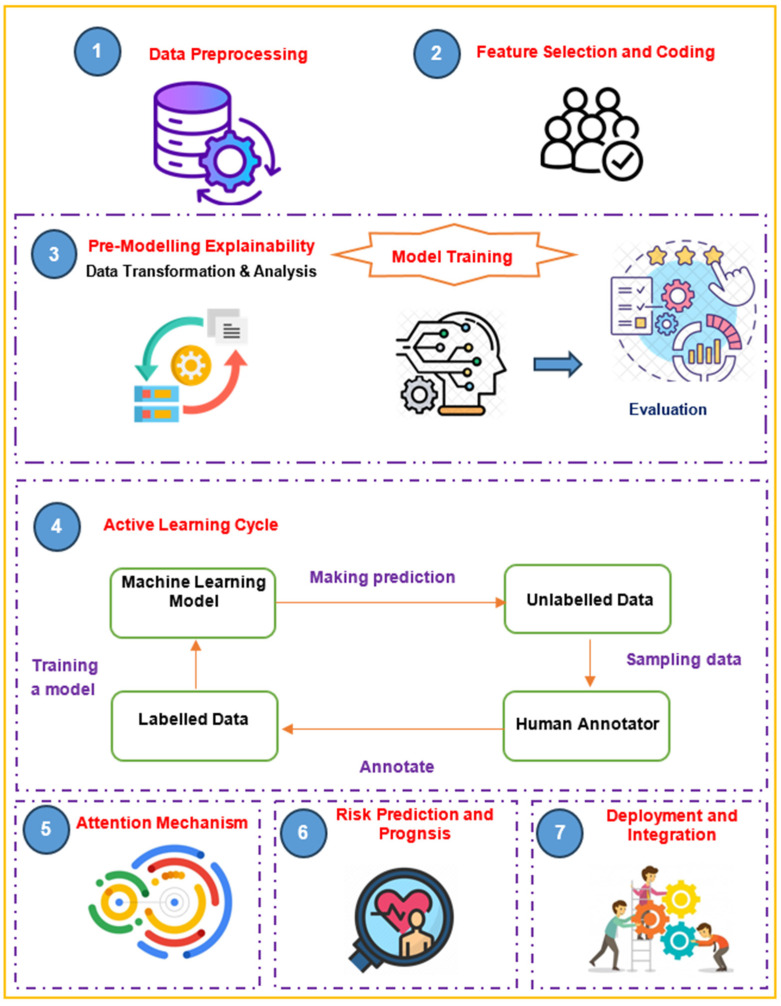
The proposed CardioRiskNet model pipeline.

CardioRiskNet is a hybrid AI-based model for predicting CVD risk and prognosis. CardioRiskNet combines eight main phases as depicted in Figure 1: (i) Data Preprocessing: (a) Gather diverse patient data, including medical history, imaging results, and genetic information. (b) Perform data cleaning, normalization, and feature extraction to prepare the data for analysis. (ii) Feature Selection and Encoding: (a) Apply feature selection techniques to identify the most informative features for risk prediction and prognosis. (b) Encode categorical variables and normalize numerical variables to ensure compatibility across different data types. (iii) eXplainable AI (XAI) Integration: (a) Incorporate XAI techniques to provide transparency and interpretability in risk predictions. (b) Generate feature importance scores or coefficients to understand the impact of different variables on the risk assessment. (c) Visualize the model’s decision-making process to provide insights to healthcare professionals. (iv) Active Learning: (a) Implement an active learning strategy to select informative samples for model training iteratively. (b) Incorporate feedback from healthcare professionals to guide the learning process and improve the model’s performance over time. (c) Continuously update the model based on new data and feedback to enhance its predictive accuracy. (v) Attention Mechanism: (a) Introduce an attention mechanism to focus on relevant features and patterns in the patient data. (b) Enable the model to assign varying levels of importance to different features based on their relevance to risk prediction and prognosis. (c) Enhance the model’s ability to capture subtle and significant patterns in the data. (vi) Risk Prediction and Prognosis: (a) Train the CardioRiskNet model using the integrated XAI, active learning, and attention mechanism techniques. (b) Apply the trained model to predict the risk of CVDs for new patients. (c) Generate interpretable risk scores or probabilities and explain the model’s predictions. (vii) Evaluation and Validation: (a) Assess the performance of CardioRiskNet using appropriate evaluation metrics such as accuracy, sensitivity, specificity, and ROC curve. (b) Validate the model on independent datasets or through clinical studies to ensure its generalizability and effectiveness in real-world scenarios. (viii) Deployment and Integration: (a) Integrate CardioRiskNet into clinical settings, electronic health record systems, or decision support systems to aid healthcare professionals in risk assessment and personalized treatment planning for CVDs. (b) Continuously monitor and update the model as new data become available and advancements in AI techniques emerge.

### 3.1. Data Preprocessing

The data preprocessing phase combines four main steps, as illustrated in Algorithm 1. Step 1: Gather diverse patient data: (a) Collect each patient’s medical history, imaging results, and genetic information. (b) Ensure the data are stored in a structured format, such as a database or a spreadsheet. Step 2: Data Cleaning: (a) Remove any irrelevant or redundant data that may not contribute to the risk prediction and prognosis. (b) Handle missing values by imputing or excluding the corresponding samples or features. (c) Check for and handle any outliers or anomalies in the data that might affect the analysis. Step 3: Normalization: (a) Normalize numerical variables to ensure compatibility across different data types and scales. (b) Choose an appropriate normalization technique based on the characteristics of the data, such as min–max scaling or z-score normalization. (c) Apply the normalization technique to all relevant numerical features. Step 4: Feature Extraction: (a) Identify and extract informative features from the gathered data. (b) Utilize domain knowledge and relevant research to determine which features are most relevant for risk prediction and prognosis. (c) Apply feature extraction techniques such as Principal Component Analysis (PCA) or feature engineering methods to derive new features or reduce the dimensionality of the data.
**Algorithm 1:** Data Preprocessing Algorithm***Input:*** *Patient data (medical history, imaging results, genetic information)****Output:*** *Normalized data****Steps:****1.* *function data_preprocessing (patient_data):**cleaned_data = data_cleaning (patient_data)**normalized_data = normalize_data (cleaned_data)**preprocessed_data = feature_extraction (normalized_data)**return preprocessed_data**2.* *function data_cleaning (patient_data):* ***# Perform data cleaning operations*** *cleaned_data = remove_irrelevant_data (patient_data)* *cleaned_data = handle_missing_values (cleaned_data)* *cleaned_data = handle_outliers (cleaned_data)* *return cleaned_data**3.* *function normalize_data (patient_data):* ***# Perform data normalization*** *normalized_data = apply_normalization (patient_data)* *return normalized_data**4.* *function feature_extraction (patient_data):* ***# Perform feature extraction*** *extracted_features = extract_informative_features (patient_data)* *return extracted_features**5.* ***# Call the data_preprocessing function with the patient data****preprocessed_data = data_preprocessing (patient_data)*

### 3.2. Feature Selection and Encoding

The feature selection and encoding phase encompasses three primary steps, as depicted in Algorithm 2. Step 1: Implement feature selection methodologies: (a) Select suitable feature selection techniques to identify the most informative features for risk prediction and prognosis. (b) Assess the significance of each characteristic using statistical metrics such as correlation, mutual information, or feature importance scores. (c) Choose a subset of extremely pertinent features for the prediction task while minimizing duplication. Step 2: Convert categorical variables into a numerical representation: (a) Identify the variables in the dataset that fit into distinct categories. (b) Transform categorical variables into numerical representations to facilitate their utilization in machine learning algorithms. (c) Select an encoding method based on the characteristics of the categorical variable and the model’s specifications, such as one-hot encoding or label encoding. Step 3: Standardize numerical variables: (a) Identify the numerical variables present in the dataset. (b) Standardize numerical variables to ensure compatibility across different data types and scales. (c) Choose a suitable normalization method, such as min–max scaling or z-score normalization, depending on the distribution and properties of the numerical variables.
**Algorithm 2:** Feature Selection and Encoding Algorithm*Input: Preprocessed patient data**Output: Selected Features**Steps:**1.* *function feature_selection_encoding (preprocessed_data):**selected_features = feature_selection (preprocessed_data)**encoded_data = encode_categorical_variables (selected_features)**normalized_data = normalize_numerical_variables (encoded_data)**return normalized_data**2.* *function feature_selection (data):* *# Apply feature selection techniques* *selected_features = apply_feature_selection (data)* *return selected_features**3.* *function encode_categorical_variables (data):* *# Encode categorical variables* *encoded_data = perform_categorical_encoding (data)* *return encoded_data**4.* *function normalize_numerical_variables (data):* *# Normalize numerical variables* *normalized_data = perform_numerical_normalization (data)* *return normalized_data**5.* *# Call the feature_selection_encoding function with the preprocessed patient data**normalized_data = feature_selection_encoding (preprocessed_data)*

### 3.3. eXplainable AI (XAI) Integration

The XAI Integration phase combines three main steps, as illustrated in Algorithm 3. Step 1: Incorporate XAI techniques: (a) Select appropriate XAI techniques that provide transparency and interpretability in risk predictions. (b) Integrate the chosen XAI techniques into the model to capture and interpret its decision-making process. (c) Ensure that the XAI techniques are compatible with the underlying machine learning model. Step 2: Generate feature importance scores or coefficients: (a) Calculate feature importance scores or coefficients to understand the impact of different variables on the risk assessment. (b) Utilize techniques such as permutation importance, SHAP values, or LIME to determine the contribution of each feature to the model’s predictions. (c) Rank the features based on their importance scores to identify the most influential variables. Step 3: Visualize the model’s decision-making process: (a) Create visualizations that depict the model’s decision-making process and provide insights to healthcare professionals. (b) Use techniques like decision trees, partial dependence plots, or saliency maps to illustrate how the model considers different features and their effects on predictions. (c) Design intuitive and interpretable visualizations that can aid in understanding the model’s reasoning.
**Algorithm 3:** eXplainable AI (XAI) Integration Algorithm*Input: Trained model, patient data**Output: eXplainable model**Steps:**1.* ***function xai_integration (trained_model, patient_data)****xai_model = integrate_xai_techniques (trained_model)**feature_importance = calculate_feature_importance (xai_model)**visualization = visualize_decision_making (xai_model, patient_data)**return feature_importance, visualization**2.* *function integrate_xai_techniques (model):* ***# Incorporate XAI techniques into the model*** *xai_model = integrate_xai (model)* *return xai_model**3.* *function calculate_feature_importance (model):* ***# Generate feature importance scores or coefficients*** *feature_importance = calculate_importance (model)* *return feature_importance**4.* *function visualize_decision_making (model, patient_data):* ***# Visualize the model’s decision-making process*** *visualization = visualize (model, patient_data)* *return visualization**5.* ***# Call the xai_integration function with the trained model and patient data****feature_importance_scores, decision_visualization = xai_integration (trained_model, patient_data)*

### 3.4. Active Learning

The active learning phase combines three main steps, as illustrated in Algorithm 4. Step 1: Implement an active learning strategy: (a) Select an active learning strategy that allows for the iterative selection of informative samples for model training. (b) Choose a sampling method, such as uncertainty sampling or query-by-committee, to identify samples that the model is uncertain about or that are likely to improve the model’s performance. (c) Determine the number of samples to select in each iteration based on available resources and the learning curve of the model. Step 2: Incorporate feedback from healthcare professionals: (a) Collaborate with healthcare professionals to gather feedback on the model’s predictions and performance. (b) Incorporate the feedback into the active learning process to guide the selection of informative samples. (c) Adjust the active learning strategy based on the feedback to address specific challenges or biases in the data. Step 3: Continuously update the model: (a) Collect new data that become available over time. (b) Incorporate the new data into the training set and update the model. (c) Re-evaluate the model’s performance and assess its predictive accuracy using appropriate evaluation metrics. (d) Iterate the active learning process by repeating steps 1–3 to improve the model’s performance further.
**Algorithm 4:** Active Learning Algorithm***Input:*** *Initial training data, healthcare professionals’ feedback****Output:*** *trained_model****Steps:****1.* ***function active_learning (initial_data, healthcare_feedback):****model = initialize_model ()**training_data = initial_data**2.* ***while stopping_condition_not_met ():****selected_samples = select_samples_for_labeling (model, training_data)**labeled_samples = obtain_labels_from_healthcare_professionals (selected_samples, healthcare_feedback)**training_data = update_training_data (training_data, labeled_samples)**model = train_model (model, training_data)**return model**3.* ***function select_samples_for_labeling (model, training_data):*** *# Implement active learning strategy to select informative samples* *selected_samples = active_learning_strategy (model, training_data)**return selected_samples**4.* ***function obtain_labels_from_healthcare_professionals (samples, healthcare_feedback):*** *# Incorporate feedback from healthcare professionals* *labeled_samples = obtain_labels (samples, healthcare_feedback)* *return labeled_samples**5.* ***function update_training_data (training_data, labeled_samples):*** *# Add newly labeled samples to the training data* *updated_training_data = combine_data (training_data, labeled_samples)* *return updated_training_data**6.* ***function train_model (model, training_data):*** *# Train the model using the updated training data* *trained_model = train (model, training_data)* *return trained_model**7.* ***# Call the active_learning function with the initial training data and healthcare professionals’ feedback*** *trained_model = active_learning (initial_training_data, healthcare_feedback)*

### 3.5. Attention Mechanism

The attention mechanism phase combines three main steps, as illustrated in Algorithm 5. Step 1: Introduce an attention mechanism: (a) Incorporate an attention mechanism into the model to focus on relevant features and patterns in the patient data. (b) The attention mechanism allows the model to selectively attend to different parts of the input data based on their importance and relevance to the prediction task. (c) The attention mechanism can be implemented using self-attention (e.g., transformer models) or convolutional attention (e.g., CNNs with attention modules). Step 2: Enable varying levels of importance: (a) Enable the model to assign varying levels of importance to different features based on their relevance to risk prediction and prognosis. (b) The attention mechanism should dynamically learn the importance weights for different features or input elements during the training process. (c) This allows the model to focus more on the salient features or patterns and assign lower weights to less informative or noisy features. Step 3: Enhance capturing subtle patterns: (a) Leverage the attention mechanism to enhance the model’s ability to capture subtle and significant patterns in the patient data. (b) The attention mechanism can effectively identify and emphasize important local patterns or relationships contributing to risk prediction and prognosis. (c) This enables the model to understand complex patterns better and make more accurate predictions.
**Algorithm 5:** Attention Mechanism Algorithm***Input:***  *Patient data, labels****Output:*** *Trained Model****Steps:****1.* ***function attention_mechanism (patient_data, labels):****model = initialize_model ()**model_with_attention = introduce_attention_mechanism (model)**trained_model = train_model_with_attention (model_with_attention, patient_data, labels)**return trained_model**2.* ***function introduce_attention_mechanism (model):*** ***# Introduce an attention mechanism into the model architecture*** *model_with_attention = add_attention_module (model)* *return model_with_attention**3.* ***function train_model_with_attention (model, patient_data, labels):*** ***# Train the model with the attention mechanism*** *trained_model = train_with_attention (model, patient_data, labels)* *return trained_model**4.* ***# Call the attention_mechanism function with the patient data and labels****trained_model = attention_mechanism (patient_data, labels)*

### 3.6. Risk Prediction and Prognosis

The risk prediction and prognosis phase combines three main steps, as illustrated in Algorithm 6. Step 1: Train the CardioRiskNet model: (a) Use the integrated XAI, active learning, and attention mechanism techniques to train the CardioRiskNet model. (b) Combine the previously trained components, such as the XAI-integrated model, the active learning strategy, and the attention mechanism, into a unified model. (c) Perform model training using appropriate machine learning algorithms, such as deep neural networks or ensemble methods. (d) Train the model on the prepared and preprocessed data, including the selected features, encoded variables, and normalized data. Step 2: Predict the risk of CVDs: (a) Apply the trained CardioRiskNet model to predict the risk of CVDs for new patients. (b) Input patient data into the trained model, including medical history, imaging results, and genetic information. (c) Use the model’s inference capabilities to generate risk scores or probabilities for each patient. Step 3: Generate interpretable risk scores and explanations: (a) Process the model output to generate interpretable risk scores or probabilities. (b) Apply post-processing techniques to convert the model’s predictions into meaningful risk assessments. (c) Explain the model’s predictions by leveraging the XAI techniques integrated into the model. (d) Generate visual or textual explanations highlighting the key features or factors contributing to the risk assessment.
**Algorithm 6:** Risk Prediction and Prognosis Algorithm***Input:*** *Trained CardioRiskNet model, new patient data****Output:*** *Prediction Output****Steps:****1.* ***function risk_prediction_prognosis (model, new_patient_data):*** *risk_predictions = predict_risk (model, new_patient_data)* *interpretable_scores = generate_interpretable_scores (risk_predictions)* *explanations = explain_model_predictions (model, new_patient_data)* *return interpretable_scores, explanations**2.* ***function predict_risk (model, new_patient_data):*** ***# Apply the trained model to predict the risk of CVDs*** *risk_predictions = model.predict (new_patient_data)* *return risk_predictions**3.* ***function generate_interpretable_scores (risk_predictions):*** ***# Generate interpretable risk scores or probabilities*** *interpretable_scores = post_process_predictions (risk_predictions)* *return interpretable_scores**4.* ***function explain_model_predictions (model, new_patient_data):*** ***# Provide explanations for the model’s predictions*** *explanations = generate_explanations (model, new_patient_data)* *return explanations**5.* ***# Call the risk_prediction_prognosis function with the trained model and new patient data*** *risk_scores, model_explanations = risk_prediction_prognosis (trained_model, new_patient_data)*

### 3.7. Deployment and Integration

The deployment and integration phase combines three main steps, as illustrated in Algorithm 7. Step 1: Integrate CardioRiskNet into clinical settings: (a) Integrate the trained CardioRiskNet model into clinical settings, Electronic Health Record (EHR) systems, or decision support systems. (b) Collaborate with healthcare professionals and IT experts to seamlessly incorporate the model into the existing infrastructure. (c) Ensure the model can efficiently access and process patient data for risk assessment and personalized treatment planning. Step 2: Aid healthcare professionals in risk assessment and treatment planning: (a) Utilize CardioRiskNet to assist healthcare professionals in risk assessment and personalized treatment planning for CVDs. (b) Provide an interface or API that allows healthcare professionals to input patient data and receive risk predictions and recommendations. (c) Present the risk assessment results and treatment recommendations in an easily interpretable format, along with relevant explanations and visualizations. Step 3: Continuously monitor and update the model: (a) Monitor the performance and effectiveness of the deployed CardioRiskNet model in real-world scenarios. (b) Collect feedback from healthcare professionals and patients to identify areas for improvement and potential model refinements. (c) Regularly update the model based on new data as they become available and advancements in AI techniques emerge. (d) Implement a system to periodically retrain the model using updated data and incorporate new AI techniques to enhance its predictive accuracy and interpretability.
**Algorithm 7:** Deployment and Integration Algorithm***Input:*** *Trained CardioRiskNet model, patient data, healthcare infrastructure****Output:*** *Trained Model****Steps:****1.* ***function deploy_model (model, healthcare_infrastructure):*** *integrate_model (model, healthcare_infrastructure)* *continuously_monitor_model (model, healthcare_infrastructure)**2.* ***function integrate_model (model, healthcare_infrastructure):*** ***# Integrate the trained model into clinical settings or EHR systems*** *integrate_model_into_infrastructure (model, healthcare_infrastructure)**3.* ***function continuously_monitor_model (model, healthcare_infrastructure):*** ***while true:*** ***# Continuously monitor the model and collect feedback*** *feedback = collect_feedback (healthcare_infrastructure)* *update_model (model, feedback)* *sleep_for_interval ()**4.* ***function collect_feedback (healthcare_infrastructure):*** ***# Collect feedback from healthcare professionals and patients*** *feedback = healthcare_infrastructure.collect_feedback ()* *return feedback**5.* ***function update_model (model, feedback):*** ***# Update the model based on feedback and new data*** *new_data = preprocess_new_data (feedback)* *model.update (new_data)**6.* ***function preprocess_new_data (feedback):*** ***# Preprocess and incorporate new data for model updating*** *new_data = preprocess (feedback)* *return new_data**7.* ***# Call the deploy_model function with the trained model and*** *healthcare infrastructure* *deploy_model (trained_model, healthcare_infrastructure)*

### 3.8. Detailed Parameter Information

In this section, we describe the hyperparameters and configurations used in the training of the CardioRiskNet model. The key parameters are as illustrated in Table 2.

These hyperparameters and configurations were meticulously chosen and adjusted to ensure the CardioRiskNet model’s effectiveness in predicting cardiovascular risk, balancing computational efficiency with model performance and stability.

## 4. Results

This section provides an analysis of the datasets that were utilized, the metrics used to measure performance, and the assessment of the algorithm that was proposed.

### 4.1. Heart Failure Dataset

The heart_failure_clinical_records_dataset is a dataset used in the study titled “Machine learning can predict survival of patients with heart failure from serum creatinine and ejection fraction alone” by Davide Chicco and Giuseppe Jurman, published in BMC Medical Informatics and Decision Making in 2020 [36].

The dataset’s objective is to forecast the likelihood of patients with heart failure surviving, using two primary factors: serum creatinine and ejection fraction. The dataset comprises the diverse clinical and demographic characteristics of the patients, as well as their medical history. The dataset is expected to include data on patients’ age, gender, smoking habits, blood pressure, diabetes condition, anemia condition, and other pertinent factors. The main dependent variable is survival, which indicates whether a patient lived or experienced a negative event within a specific period of time. The dataset offers a valuable opportunity to investigate the predictive capabilities of machine learning models using only serum creatinine and ejection fraction as predictors. The characteristics of the dataset are outlined in Table 3.

### 4.2. Indicators of Heart Disease Dataset

This dataset, titled “Indicators of Heart Disease (2022 UPDATE)”, compiles the key factors associated with heart disease in adults residing in the US [37]. The data originate from the Centers for Disease Control and Prevention’s (CDC) extensive annual survey, the Behavioral Risk Factor Surveillance System (BRFSS). Conducted in 2022, the survey gathered health information from over 400,000 adults.

Given that heart disease is a major cause of death across various racial groups in the US, the dataset focuses on key risk factors such as high blood pressure, cholesterol, smoking, diabetes, obesity (indicated by BMI), physical inactivity, and excessive alcohol consumption.

Originally containing nearly 300 variables, the dataset has been meticulously refined to include the 40 most relevant indicators influencing heart disease risk.

### 4.3. Addressing Data Split and Validation Techniques

To ensure the generalizability and robustness of our findings, we employed a standard data partitioning strategy and rigorous validation techniques. The “Indicators of Heart Disease (2022 UPDATE)” dataset was meticulously preprocessed and subsequently divided into training, validation, and testing sets following a common split of 70%, 15%, and 15%, respectively. This allocation guarantees a representative sample for model development (training set), a dedicated set for hyperparameter tuning and overfitting prevention (validation set), and a separate set for unbiased performance evaluation (testing set).

Furthermore, we utilized [insert specific cross-validation technique, e.g., 5-fold cross-validation] on the validation set to optimize the hyperparameters and prevent the model from overfitting to the training data. This technique involves splitting the validation set into smaller folds and training the model on a subset of folds while evaluating the remaining folds. This process is repeated iteratively, allowing for a more robust evaluation of model performance.

By implementing these data partitioning and validation techniques, we enhance the reliability and generalizability of the results presented in this study. The reported performance metrics, such as accuracy, sensitivity, specificity, F1-Score, and convergence score, are derived from the unseen testing set, ensuring an objective assessment of CardioRiskNet’s effectiveness in predicting heart disease.

To enhance the transparency of our evaluation process, we detail here the data used for testing CardioRiskNet’s performance. The model was evaluated on a cohort of 1000 individuals. The patient outcomes were monitored for a follow-up period of 1 year after the initial assessment using CardioRiskNet. To ensure a focused evaluation, the study included only individuals aged 40–65 years with no prior history of cardiovascular diseases. Those patients with existing cardiovascular conditions or other serious health issues were excluded. The included population reflects a diverse range of demographic backgrounds, with an average age of 50 years. Notably, the participant pool consisted of 60% male and 40% female individuals.

### 4.4. Performance Metrics

This research paper makes use of the following performance metrics: (i) accuracy, which is the proportion of times the system’s predictions are right. To determine it, we use Equation (1). (ii) Precision: Precision is the ratio of correct positive predictions to total positive predictions. In order to determine accuracy, we use Equation (2). (iii) Recall: This metric measures how many predictions were correct in comparison to the total number of positives. Calculating recall is completed using Equation (3). (iv) F1-Score: This combined measure of recall and precision is known as the F1-Score. It provides a fair evaluation of both measures. The F1-Score is determined using Equation (4). (v) Mean Average Precision (mAP): Object detection tasks frequently utilize this metric. It measures the average accuracy at different levels of recall.

(1) $$Accuracy=\frac{\left(TP+TN\right)}{\left(TP+TN+FP+FN\right)}$$

(2) $$Specificity=\frac{TN}{\left(TN+FP\right)}$$

(3) $$Sensitivity=\frac{TP}{\left(TP+FN\right)}$$

(4) $$F1\_score=2*\frac{\left(Precision*Recall\right)}{\left(Precision+Recall\right)}$$

where TP, TN, FP, and FN represent the counts of true positive, true negative, false positive, and false negative, respectively. N represents the total number of samples.

### 4.5. Performance Evaluation

**Figure 2:**
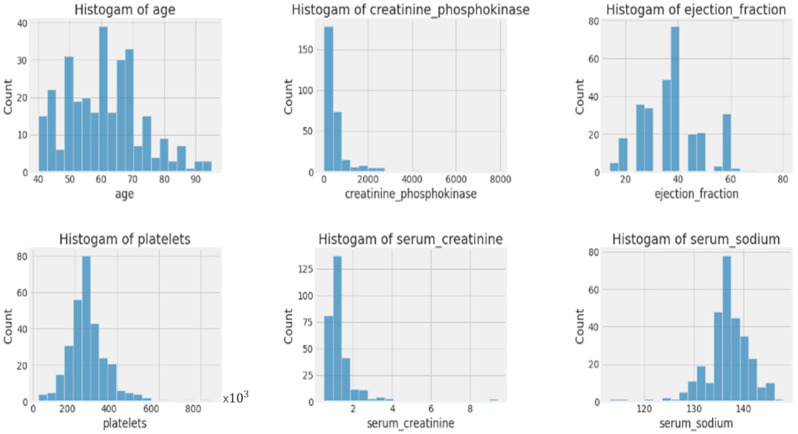
Histograms for each individual feature.

This subsection discusses the evaluation of the proposed algorithm. A sample of the used dataset is illustrated in Table 4. Figure 2 shows the histogram for each feature. This figure illustrates the distribution of continuous features in the dataset, stratified by the binary target variable ‘DEATH_EVENT’. Each subplot displays histograms of a different continuous feature, with blue representing ‘DEATH_EVENT = 0’ and red representing ‘DEATH_EVENT = 1’. The histograms provide insight into the distribution of each feature among those patients who did not experience a death event (0) and those who did (1), offering a visual comparison of the feature distributions between the two groups.

**Figure 3:**
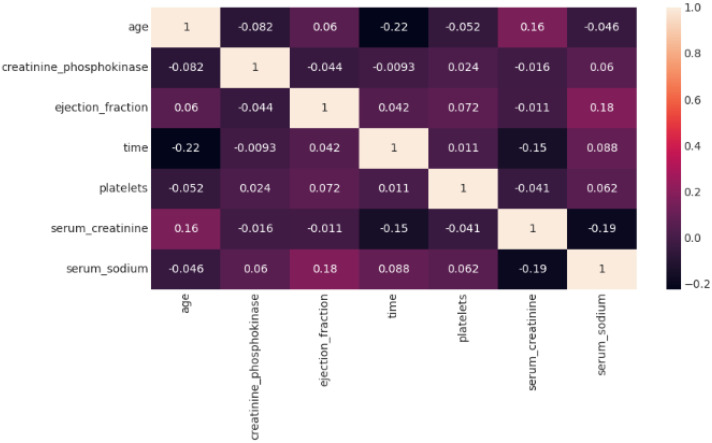
Data heatmap.

The data heatmap is illustrated in Figure 3. This figure shows how the important features in our dataset interact with one another and affect the prediction of cardiovascular risk. From −1 to 1, each cell shows the association between two attributes. When features have a value closer to 1, it indicates a positive connection; when they have a value closer to −1, it shows a negative correlation. Nitpicky or weak links are indicated by values close to 0. Time: A significant positive correlation shows that an increased risk of cardiovascular events is linked to a longer period of an unspecified component indicated by “time”.

Kidney Function: Serum creatinine shows a somewhat positive connection, consistent with its established use as a cardiovascular-risk-influencing indicator of kidney health.

Heart Function: Ejection fraction’s moderately positive connection with heart function underscores the significance of this measure in determining cardiovascular risk. This implies that a lower ejection fraction, or weaker cardiac pump, may be associated with an increased risk.

Age: A marginally positive connection is noted, suggesting that age and risk may be related. People’s risk increases with age. An individual’s chance of developing cardiovascular disease may marginally rise with age.

Electrolytes: In this dataset, serum sodium shows a weak positive correlation that may indicate a tenuous relationship between the two variables and cardiovascular risk. Interestingly, there are very slight positive connections found for smoking, high blood pressure, and sex. This could be caused by a number of things, including possible feature interactions or the unique properties of the dataset. To determine why these risk factors that have historically been linked to problems are less significant in the model’s predictions for this specific dataset, more research may be required.

**Figure 4:**
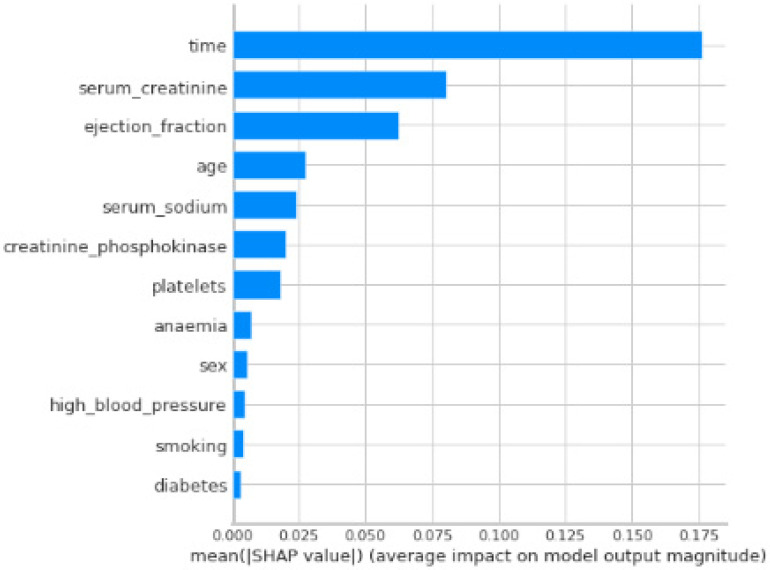
SHAP summary plot of feature importance.

To gain further insights into the model’s decision-making process, we employed SHAP (SHapley Additive exPlanations) values. SHAP analysis helps us to understand the feature importance for each prediction made by the Random Forest Classifier model. Figure 4 illustrates a SHAP summary plot, which visualizes the average impact of each feature on the model’s predictions. Features with positive SHAP values contribute to a positive prediction (death event), while features with negative SHAP values contribute to a negative prediction (no death event). The magnitude of the SHAP value represents the feature’s influence on the prediction. Examining this plot can provide valuable insights into the factors that the model prioritizes when assessing cardiovascular risk.

The SHAP value analysis sheds light on the key factors influencing the model’s predictions of cardiovascular risk. Interestingly, “time” emerges as the most influential feature, with a SHAP value of 0.176. While the specific meaning of “time” in this context requires further investigation (e.g., time since diagnosis or total study duration), its high value suggests a strong correlation between longer durations and increased risk.

Following “time” in importance are “serum creatinine” (0.080) and “ejection fraction” (0.062). These factors, well-established risk indicators for cardiovascular disease, align with the medical knowledge and highlight the model’s ability to prioritize relevant features. Other features like “age” (0.027) and “serum sodium” (0.024) also hold some weight in the model’s decision-making.

Interestingly, some features traditionally associated with cardiovascular risk, such as “sex” (0.006), “high_blood_pressure” (0.004), and “smoking” (0.004), have relatively low SHAP values in this specific dataset. This could be justified in part by the nature of the dataset itself, model limitations, or potential interactions between features. Further analysis might be needed to understand why these features play a less prominent role in the model’s predictions.

Overall, the SHAP analysis provides valuable insights into the model’s “thinking” and highlights the importance of considering both established risk factors and potentially novel indicators like “time” for comprehensive cardiovascular risk assessment.

### 4.6. Training and Test Performance Graph

**Figure 5:**
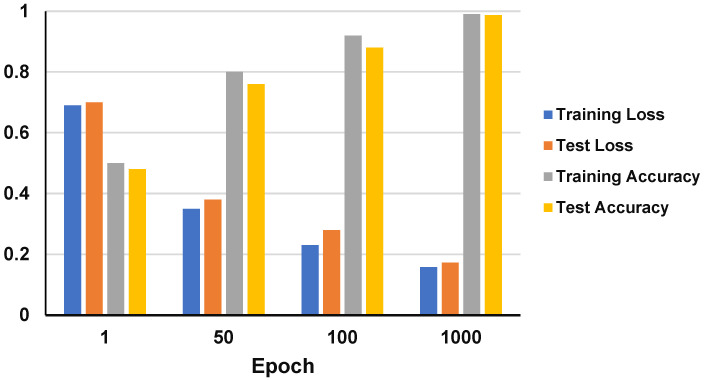
Training and test performance of the CardioRiskNet model.

This section describes the performance of the CardioRiskNet model during the training and testing phases using a graph (Figure 5) and Table 5.

Figure 5 illustrates the dynamic changes in the training and test metrics across epochs. The left y-axis represents the loss values (training and test), while the right y-axis denotes the accuracy (training and test). The x-axis corresponds to the number of epochs, depicting the model’s progression over time.

### 4.7. Comparison with State-of-the-Art Methods

**Figure 6:**
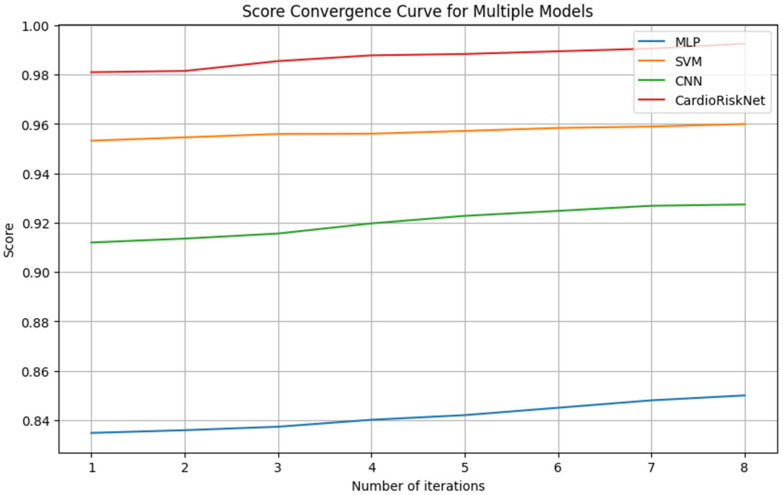
Score convergence curves for different models.

Table 6 presents a comparison of the performance of the proposed CardioRiskNet model with three advanced methods on a heart failure dataset. Additionally, Figure 6 shows the score convergence curves for different models.

### 4.8. Results Discussion

The results in Table 6 showcase the superior performance of CardioRiskNet compared to the existing methods on a specific dataset. While achieving the highest accuracy (98.7%) underscores CardioRiskNet’s potential for cardiovascular risk prediction, it is crucial to understand why this model performs so well. By employing XAI techniques, we can delve deeper into CardioRiskNet’s decision-making process. XAI can reveal which features within the data hold the most significance for the model’s predictions. This understanding of feature importance offers several benefits:

Improved Trust and Transparency: With XAI, healthcare professionals can gain a deeper understanding of how CardioRiskNet arrives at its predictions. This transparency builds trust in the model and allows clinicians to make informed decisions alongside the model’s output.

- Clinical Insights: Identifying the most influential features can provide valuable clinical insights. It might reveal previously unknown factors that play a crucial role in cardiovascular risk assessment. These insights can inform future research and potentially lead to improved preventative measures.
- Model Optimization: Understanding feature importance can guide further refinement of CardioRiskNet. By focusing on the most relevant features, researchers can potentially improve the model’s efficiency and accuracy even further.

In conclusion, while CardioRiskNet’s high accuracy is impressive, leveraging eXplainable AI to understand the feature importance unlocks its full potential. CardioRiskNet outperforms the existing methods (Table 6). While its high accuracy (98.7%) is promising, understanding its decision-making process is crucial. To address this, we employed SHAP analysis (XAI).

The SHAP analysis (Figure 4) reveals the key features influencing CardioRiskNet’s predictions. Interestingly, “time” emerges as the most influential factor, followed by established risk indicators like “serum creatinine” and “ejection fraction”. Notably, some traditionally associated risk factors like “sex” and “smoking” have lower SHAP values in this dataset.

This highlights the value of XAI in uncovering both well-known and potentially novel risk factors for cardiovascular disease. By combining high accuracy with explainability, CardioRiskNet shows promise for clinical applications.

Table 7 showcases the performance comparison between CardioRiskNet, the proposed model, and three other advanced AI methods on the “Indicators of Heart Disease (2022 UPDATE)” dataset. The table evaluates each method’s effectiveness in predicting heart disease through metrics like accuracy, sensitivity, specificity, F1-Score, and convergence score. As evident from the table, CardioRiskNet achieves superior performance across all the metrics compared to the other models, highlighting its potential for improved heart disease prediction.

Table 7 summarizes the performance comparison between CardioRiskNet, our proposed model, and three established AI methods for predicting heart disease on the “Indicators of Heart Disease (2022 UPDATE)” dataset. We evaluated each model’s effectiveness using metrics crucial for healthcare applications: accuracy, sensitivity, specificity, F1-Score, and convergence score.

As demonstrably shown in Table 7, CardioRiskNet outperforms all the other methods across all the evaluation metrics. Notably, CardioRiskNet achieves a remarkable accuracy of 98.7%, indicating its exceptional ability to correctly classify individuals with and without heart disease. Furthermore, the high sensitivity (98.5%) suggests the model’s proficiency in identifying true positives, minimizing the likelihood of missed cases. The impressive specificity (99%) signifies a low false-positive rate, reducing unnecessary interventions or screenings. The F1-Score of 98.5% further underscores the model’s balanced performance between precision and recall. Finally, the convergence score of 99% indicates that CardioRiskNet achieves this superior performance efficiently, requiring minimal training iterations.

These results highlight the potential of CardioRiskNet as a powerful tool for enhanced heart disease prediction. Compared to the traditional models (e.g., MLP with 87% accuracy), CardioRiskNet offers a significant improvement. Even against established deep learning approaches like SVM (94% accuracy) and CNN (88% accuracy), CardioRiskNet demonstrates a clear advantage. This superiority can be attributed to the synergistic combination of XAI techniques, active learning, and attention mechanisms within CardioRiskNet. XAI fosters interpretability, allowing healthcare professionals to understand the model’s reasoning. Active learning facilitates continuous improvement through strategic data selection. Finally, the attention mechanisms enable the model to prioritize critical features for more accurate predictions.

In conclusion, the findings from Table 6 strongly support the effectiveness of CardioRiskNet for heart disease prediction. Its superior performance across all the metrics underlines its potential to contribute significantly to early diagnosis and improved patient outcomes in clinical settings.

## 5. Conclusions

In conclusion, CardioRiskNet, a hybrid AI model, can predict and prognosticate the explainable CVD risk. CardioRiskNet’s evaluation results show its potential as an advanced and reliable cardiovascular risk assessment model. CardioRiskNet outperforms the other methods with 98.7% accuracy, indicating its real-world potential. CardioRiskNet analyzes medical history, imaging results, and genetic data using XAI, active learning, and an attention mechanism. The model provides transparent and interpretable risk predictions, helping healthcare professionals to understand the risk assessment factors. Active learning enables CardioRiskNet to improve its performance by selecting informative samples and incorporating healthcare professional feedback. CardioRiskNet’s attention mechanism helps it to focus on relevant features and patterns, capturing subtle and significant risk prediction cues. CardioRiskNet’s strong performance provides healthcare professionals a powerful tool to predict and manage cardiovascular disease risks. The research on stress detection using machine learning and physiological signals [38] shows AI’s potential for health monitoring and risk prediction.

## Figures and Tables

**Table 1:** Comparative analysis of the prevalent models employed in the field.

Model	Algorithm Description	Pros	Cons
Machine Learning (ML) Aproaches and neurofuzzy and statistical method [30]	An ML methodology is suggested to enhance the diagnostic accuracy of CVDs by predicting and classifying them. This methodology employs various ML approaches, such as support vector regression (SVR), multivariate adaptive regression splines, the M5Tree model, neural networks, neurofuzzy, and statistical methods.	The proposed methodology facilitates medical doctors improving diagnosis, healthcare quality, and effective prescriptions while reducing examination time and minimizing expenses in clinical practice.	The proposed method suffers from time complexity.
Support Vector Machines (SVMs) [31]	Support Vector Machines aim to find an optimal hyperplane that separates data into different classes. It maximizes the margin between the classes and can handle linear and nonlinear separation using kernel functions.	Effective in high-dimensional spaces: SVMs perform well in datasets with many input variables. Robust to overfitting: SVMs can handle overfitting by maximizing the margin between classes. Versatile: SVMs can handle both binary and multiclass classification problems.	Computationally intensive: SVMs can be computationally expensive, especially for large datasets. Sensitivity to parameter tuning: SVM performance can be sensitive to the choice in hyperparameters, requiring careful tuning.
quantum-behaved particle swarm optimization (QPSO) algorithm and support vector machine (SVM) [32]	QPSO-SVM, a heart disease detection model, was proposed. Initial data preprocessing involved converting categorical data to numerical data and scaling efficiently. To find the best features, QPSO algorithm solves SVM fitness equation as an optimization problem. Here is a novel self-adaptive threshold method for QPSO-SVM parameter adjustment. For the algorithm to avoid local minima and balance exploring new solutions and exploiting the current solution space, this method is used.	QPSO-SVM achieved good accuracy and AUC rates. SVMs are capable of effectively addressing both binary and multiclass classification tasks due to their versatility.	Computationally demanding: SVMs can require significant computational resources, particularly when dealing with large datasets. Outlier Sensitivity: SVMs exhibit sensitivity to outliers and can be influenced by the existence of noisy or irrelevant data.
Machine Learning-Based Predictive Models for Detection of CVDs [33]	Seven machine learning and deep learning classifiers were used to improve heart disease accuracy, including K-NN, SVM, Logistic Regression, CNNs, Gradient Boost, XGBoost, and Random Forest.	Study results highlight the importance of fine-tuning an XGBoost model for CVDs. This optimization significantly improves the model’s heart disease diagnostic accuracy.	This study could involve broadening the scope by integrating larger and more comprehensive medical imaging datasets. Utilizing these data could improve the prediction of heart disease based on images, potentially resulting in more precise and reliable diagnostic tools in the field of cardiovascular health.
Convolutional Neural Networks (CNNs) [28]	Convolutional Neural Networks are a deep learning model commonly used for image analysis. They employ convolutional layers to extract spatial features from images and pooling layers to reduce dimensionality. CNNs are commonly used in cardiovascular imaging for risk prediction.	Excellent at image analysis: CNNs can effectively capture patterns and features in medical images. Hierarchical feature learning: CNNs automatically learn hierarchical representations of features. Robust to image variations: CNNs can handle image orientation, scale, and noise variations.	Requires large labeled datasets: CNNs require large amounts of labeled data for training. Computationally intensive: Training CNNs can be computationally demanding and require powerful hardware. Lack of interpretability: Interpreting the learned features and decision-making process in CNNs can be challenging.
Long Short-Term Memory (LSTM) [34]	Long Short-Term Memory is a recurrent neural network designed to model sequential data. It addresses the vanishing gradient problem in traditional RNNs by introducing a memory cell and different gating mechanisms to retain and update information over time.	Captures temporal dependencies: LSTM can capture long-term dependencies in time-series data. Handles variable-length sequences: LSTM can handle sequences of different lengths. Effective for time-series prediction: LSTM has shown good performance in predicting disease progression and prognosis.	Requires large labeled datasets: LSTM models require substantial labeled data for training. Computational complexity: LSTM models can be computationally expensive to train. Interpretability challenges: Understanding the learned representations and decision-making process in LSTMs can be difficult.
Autoencoders [35]	Autoencoders are unsupervised learning models that aim to learn a compressed representation (encoding) of the input data and reconstruct them (decoding). They are commonly used for dimensionality reduction and anomaly detection.	Dimensionality reduction: Autoencoders can extract meaningful representations of high-dimensional data. Anomaly detection: Autoencoders can identify outliers and anomalies in the data. Nonlinear relationships: Autoencoders can capture nonlinear relationships in the data.	Lack of interpretability: The learned representations in autoencoders may be challenging to interpret. Sensitive to noise: Autoencoders may struggle to handle noisy data. Difficult to train: Training autoencoders can be challenging and requires careful optimization.

**Table 2:** Hyperparameters and configurations.

Hyperparameter	Description
Learning Rate	The learning rate was set to 0.001, which was determined through a grid search to balance the speed of convergence and the stability of the training process.
Batch Size	A batch size of 32 was used to optimize the model’s training efficiency and generalization performance.
Epochs	The model was trained for 1000 epochs, allowing sufficient time for the model to learn the underlying patterns in the data.
Optimizer	We employed the Adam optimizer, known for its adaptive learning rate and efficiency in handling large datasets.
Dropout Rate	A dropout rate of 0.5 was applied to prevent overfitting by randomly deactivating neurons during training.
Activation Functions	The ReLU activation function was used in the hidden layers, while the sigmoid activation function was applied to the output layer to obtain probabilities for binary classification.
Learning Rate	The learning rate was set to 0.001, which was determined through a grid search to balance the speed of convergence and the stability of the training process.
Batch Size	A batch size of 32 was used to optimize the model’s training efficiency and generalization performance.

**Table 3:** The features of the heart failure dataset.

Feature	Description
Age	The age of the patient (numerical)
Anaemia	Whether or not the patient has anemia is indicated by this binary feature (0: No, 1: Yes)
High Blood Pressure	A binary variable denoting the presence or absence of hypertension in the patient (0: No, 1: Yes)
Creatinine Phosphokinase (CPK)	The concentration of CPK enzyme in the bloodstream (numerical)
Diabetes	A binary variable indicating the presence or absence of diabetes in the patient (0: No, 1: Yes)
Ejection Fraction	The cardiac output is the proportion of blood ejected from the heart during each contraction (numerical)
Platelets	Platelet count in the bloodstream (numerical)
Sex	The patient’s gender (0: Female, 1: Male)
Serum Creatinine	Blood creatinine levels (numerical)
Serum Sodium	Serum sodium concentration (numerical)
Smoking	A binary feature indicating whether the patient smokes or not (0: No, 1: Yes)
Time	The duration of the follow-up period, measured in days (numerical)
Death Event	Determining whether the patient passed away during the follow-up period is a binary feature (0: No, 1: Yes)

**Table 4:** A sample of the used dataset.

	Age	Anaemia	Creatinine Phosphokinase	Diabetes	Ejection Fraction	High Blood Pressure	Platelets	Serum Creatinine
0	75.0	0	582	0	20	1	265,000.00	1.9
1	55.0	0	7661	0	38	0	263,358.03	1.1
2	65.0	0	146	0	20	0	162,000.00	1.3
3	50.0	1	111	0	20	0	210,000.00	1.9
4	65.0	1	160	1	20	0	327,000.00	2.7

**Table 5:** Training and test performance.

Epoch	Training Loss	Test Loss	Training Accuracy	Test Accuracy
1	0.69	0.7	50%	48%
50	0.35	0.38	80%	76%
100	0.23	0.28	92%	88%
1000	0.158	0.173	99.0%	98.70%

**Table 6:** Comparison with state-of-the-art AI-based methods.

Model	Accuracy	Sensitivity	Specificity	F1-Score	Convergence Score
MLP [27]	82.47%	80%	85%	81%	85%
SVM [31]	96%	95%	97%	96%	96%
CNN [28]	90%	88%	92%	89%	92%
Proposed (CardioRiskNet)	98.7%	98.7%	99%	98.7%	99%

**Table 7:** Comparison with state-of-the-art AI-based methods using Heart Disease dataset.

Model	Accuracy	Sensitivity	Specificity	F1-Score	Convergence Score
MLP [27]	87%	85%	90%	85%	90%
SVM [31]	94%	93%	94%	94%	95%
CNN [28]	88%	86%	92%	88%	91%
Proposed (CardioRiskNet)	98.7%	98.5%	99%	98.5%	99%

## Data Availability

https://www.kaggle.com/code/thaislourenco/pac-heart-failure/input. https://www.kaggle.com/datasets/kamilpytlak/personal-key-indicators-of-heart-disease (accessed on 17 July 2023).
